# Comment on “Utility of a Novel 12‐Lead ECG Criterion for Localizing Manifest Right‐Sided Accessory Pathways: A Derivation Study”

**DOI:** 10.1002/joa3.70338

**Published:** 2026-04-10

**Authors:** Sidra Afzal, Nimra Afzal, Sana Afzal

**Affiliations:** ^1^ Fatima Jinnah Medical University Lahore Pakistan; ^2^ Anglia Ruskin University Cambridge UK; ^3^ University of Veterinary and Animal Sciences Lahore Pakistan

To the Editor,

We read with great interest the recent study proposing the PR/QRS ratio as a new ECG criterion for locating manifested right‐sided accessory pathways (APs) [1] The effort to improve noninvasive localization of APs, especially high‐risk septal pathways, is commendable. It impacts patient counseling, procedural planning, and risk reduction.

While the study shows promising accuracy at 88.8% with a strong area under the curve of 0.878, several methodological and clinical considerations need discussion.

*1: Derivation Without External Validation*: The study used a single‐center group to derive the PR/QRS ratio. Prediction models developed in one dataset are likely to overfit, which can inflate reported accuracy. External validation in independent populations is important to see if the measure works reliably across different patient groups [2].

*2: Comparative Benchmarking*: The authors did not directly compare the PR/QRS ratio with established ECG localization algorithms, as shown in previous studies [3] It is important to determine whether the new criterion provides additional value over existing methods to evaluate its clinical usefulness.

*3: Sample Characterization*: Right‐sided APs include sub‐regions: anteroseptal, mid‐septal, and posteroseptal. The distribution of these subtypes can impact diagnostic accuracy. If some subtypes are underrepresented, the reported sensitivity and specificity might not show real‐world performance. Offering detailed subgroup data would help clarify generalizability.

*4: Inter‐observer Reproducibility*: ECG measurements, especially QRS and PR intervals, are subject to intra and inter‐observer variability. Without formal evaluation, the reliability of this criteria in daily clinical practice remains unclear.

*5: Clinical Applicability and Limitations*: One reason the PR/QRS ratio works well—hitting 88.8% accuracy—is it responds clearly to typical patterns. Yet when hearts already show damage or odd electrical signals, how it behaves remains unclear [4]. Past attempts found location predictions often miss the mark under those conditions [5]. That uncertainty means testing must come first, especially where anatomy complicates readings.

Putting it together, the PR/QRS ratio offers a fresh take on an issue doctors face every day. Tackling those earlier concerns—like testing in outside settings, how it stacks up against current methods, consistent results across trials, and its behavior in different patient groups—could make this work more likely to move into real‐world care without risk.

## Funding

The authors have nothing to report.

## Conflicts of Interest

The authors declare no conflicts of interest.

## Data Availability

Data sharing not applicable to this article as no datasets were generated or analysed during the current study.
